# Does attending general practice prior to the emergency department change patient outcomes? A descriptive, observational study of one central London general practice

**DOI:** 10.1080/17571472.2017.1280893

**Published:** 2017-01-23

**Authors:** S. Morton, R. Hames, I. Kelso, A. Newth, S. Gnani

**Affiliations:** a North Kensington Medical Centre, London, UK; b Department of Primary Care and Public Health, School of Public Health, Imperial College London, London, UK

**Keywords:** General practice, emergency department, attendances, emergency admissions

## Abstract

**Background:**

The challenge of keeping Emergency Department (ED) attendances down continues and timely access to general practice (GP) is often portrayed as a potential solution.

**Setting:**

One London general practice (registered population = 4900)

**Question:**

Does seeing a GP before attending the ED affect the outcome of a patient’s ED care?

**Methods:**

Routine clinical data were extracted using SystmOne primary care computer system for all registered patients with an ED attendance between 1 October 2014 and 31 September 2015. The scanned discharge summaries from the ED and GP notes were reviewed and outcome measures extracted.

**Results:**

227 patients (121 female; 104 male) attended the ED. The most common presentation was abdominal pain (*n* = 11). 25% of patients had seen (*n* = 50), or contacted by phone (*n* = 6), a GP about the same presenting complaint before attending the ED. Of those, 73% (*n* = 41/56) were referred to the ED and 49% (*n* = 20/41) were admitted versus 33% (*n* = 60/184) who self-presented (statistically significant, *p* = 0.05). An additional 32% of those who saw the GP first (*n* = 13/41) received specialist ED treatment.

**Discussion/Conclusion:**

Only 25% of patients see their GP prior to attending the ED. The majority of patients who were referred by their GP required admission or specialised ED treatment. It remains unclear why the majority of patients did not choose to contact their GP prior to attending the ED, despite urgent appointments being offered; research into patients’ health beliefs in this group is required for greater understanding.

## Why this matters to me

Within the media general practice opening times is often portrayed as being the answer to the growing demand on the emergency department. However, I felt that in my experience as a clinician this was normally not the case. We therefore decided to look at the outcomes of patients attending the emergency department to establish whether contact with their GP prior to attending increased the appropriateness of ED attendances.

## Key Message

The vast majority of patients attending the emergency department (ED) do not contact or see their general practitioner (GP) prior to attending, despite urgent appointments being offered morning and afternoon. Of the patients who do see their GP and are then referred into the ED a greater proportion requires admission compared to those who do not see the GP prior to ED attendance. More research is required in order to understand the barriers that stop patients accessing their GP prior to attending the ED.

## Introduction

The UK continues to experience very high demands on the emergency department (ED), with over 22 million attending the ED in England in the year 2014–2015 [1]. Within the media the rise in ED attendances continues to be well documented [2]. Alongside this incentives for general practitioners (GP) to cut ED attendances in reward for payment have also been portrayed [3].

Many studies have investigated why patients choose to attend the ED and lack of access to general practice is often cited as a reason [4,5]. It is estimated that 26.5% of unplanned ED attendances in 2012–2013 in England were due to being unable to either obtain an appointment or a convenient appointment [6]. Patients often report being very concerned about their condition and as they are unable to get an immediate appointment with their GP they decide to attend the ED [4]. One study found that the majority of adult patients did contact their GP first with an acute medical problem, although most did not actually see a GP before attending the ED [7]. However, another study showed that those who were deemed ‘inappropriate attendees’ by one emergency department used primary care services significantly more than attendees that were deemed appropriate [8]. Indeed social deprivation is recognised as one of the strongest predictors of ED attendance [9,10].

A lot of effort has been made to make access to primary care easier for patients and yet studies continue to show that access to primary care is not what determines avoidable ED attendances, and is more likely to be systemic factors such as underlying deprivation [11,12]. To the best of our knowledge, no study has looked at ED attendances from a GP specific perspective in regards to reasons for attendances and with the policy changes implemented we wanted to establish how many patients had seen a GP prior to attending ED and whether this meant they were more or less likely to be admitted. Our aim was to describe whether seeing, or contacting via phone, a GP prior to attending the ED affected the onward journey of the patient.

## Methods

### Setting

Data was collected from one North West London general practice. The general practice had 4900 patients registered in October 2015, with two full time GP partners, one part-time salaried GP and one part-time academic F2. The practice is open Monday to Friday 8 am to 6.30 pm, except for a Wednesday afternoon when the practice shuts at 1 pm. On at least two days a week appointments are offered from 7.30 am. Emergency appointments are released on the day every day, at least three every morning and three every afternoon. There is an urgent care centre within half a mile available for out of hours appointments. The GP patient survey practice report in December 2013 found that 76% of patients felt the practice was open at convenient times and 86% of patients felt it was a good experience when making an appointment [13]. There is no direct referral pathway to the medical or surgical teams within the local hospitals and so if admission is required patients must first attend the ED.

The practice falls within the Royal Borough of Kensington and Chelsea, the borough with the second highest population density in the country [14]. Despite being recognised as a relatively wealthy area, unemployment is higher than the national average [15]. Also, over 20% of residents do not speak English as their first language [15].

No ethical approval was sought for this study, in line with the National Research Ethics Service Guidance, as this project was part of a practice audit on access and was considered a service evaluation [16]. Access to the patients’ notes was granted by the partners at the practice.

### Data sources

The general practice uses SystmOne for record keeping. An analysis was run to extract all patients that had been given a Read code for attending the ED (or any other unscheduled hospital admission) between, and including, October 2014 to September 2015. All data was anonymised and recorded using Microsoft Excel.

Following extraction of the data-set one author (SM) reviewed the discharge summary from the ED for each patient coded as having attended the ED to extract the primary diagnosis and to establish whether the patient had been admitted. The primary diagnosis was then categorised into a Read code chapter [17]. If the patient had attended the ED multiple times the most recent discharge summary was used and it was recorded that they had multiple attendances within the twelve months. Using the date and time of the attendance the patients’ records were also reviewed for any notes relating to a GP or UCC attendance for the same primary diagnosis prior to attending the ED, along with documentation as to whether the patient had been referred to the ED by the GP or UCC. An ‘out of hours’ attendance was deemed anything that was outside 8 am–6.30 pm Monday to Friday. ED only treatment was deemed as anything that a GP could not offer, for example a diagnostic test or review by a speciality. Data was analysed using Microsoft Excel and SPSS statistics version 22. Chi squared tests were used for nominal data and independent *t*-tests for numerical data; significance was set at *p* = 0.05.

## Results

In total 227 of the 4900 patients registered at one central London GP surgery attended the ED between October 2014 and September 2015; 121 were female and 104 were male. Two patients were excluded as there was no discharge summary available for their attendance. The average age for attendance was 45.7 years (range 0–99 years); 65 patients (28%) were ≥ 65 years.

25% of patients had seen (*n* = 50), or contacted by phone (*n* = 6), a GP about the same primary complaint before attending the ED. Of those, 73% (*n* = 41/56) were referred to the ED by their GP. An additional 14 patients (6%) were seen by an urgent care centre doctor and referred to the ED.

The reasons for attendance according to read code are shown in Table 1. ‘Injury/poisoning’ was the most common reason for attendance, for example ‘Fracture’ (*n* = 9), followed by ‘Nervous system/senses’, which included strokes, epilepsy and eye problems such as acute angle glaucoma. The most common overall presentation to the ED was abdominal pain (*n* = 11). Head injury (*n* = 11), fracture (*n* = 9) and non-cardiac chest pain was the next most common (*n* = 8). Two patients self-discharged and were therefore excluded from the diagnostic data.

**Table 1.** Main presenting diagnosis by read chapter category.

Diagnosis (READ chapter category)	Total	Percentage
Injury/poisoning	44	19.6
Nervous system/senses	34	15.1
Digestive system	30	13.3
Respiratory system	24	10.7
Infectious/parasitic diseases	23	10.2
Circulatory system	21	9.3
Musculoskeletal	19	8.4
Genito-urinary system	14	6.2
Skin/subcutaneous tissue	4	1.8
Ill-defined conditions/working diagnoses	2	1.8
Pregnancy/childbirth/puerperium	3	1.3
Mental disorders	2	0.9
Neoplasms	1	0.4
Endocrine/metabolic	1	0.4
Blood diseases	1	0.4
Total	223*	

* 2 data sets missing due to self-discharge.

In total 36% of patients (*n* = 80/225) were admitted into hospital. 108 patients (48%) were seen in the ED ‘out of hours’, of which 38 (35%) were admitted. 64 patients (28%) had more than one ED admission recorded.

Of the 41 patients referred to the ED by their GP 49% (*n* = 21) were admitted with an additional 32% (*n* = 13/41) receiving ED only treatment. This is in comparison to 33% of patients who self-presented to ED who were admitted (*n* = 60/184), which is a statistically significant difference (*χ* = 3.83, *p* = 0.05). There was no statistically significant difference between the age of the patients who self-presented vs. those who saw the GP first (44.5 years vs. 51.1 years respectively, *p* = 0.83) nor the gender (48% male vs. 39% male respectively, *p* = 0.31) (Table 2).

**Table 2.** Breakdown of attendances to A&E.

	Seen GP first and referred on to A&E = 41	Seen GP and not referred by GP to A&E but attended A&E anyway = 9	Did not see GP = 184
Admitted by A&E	21 (49%)	3 (33%)	60 (33%)
Received A&E only treatment	13 (32%)	2 (22%)	*
Were discharged without A&E specific treatment	7 (17%)	4 (44%)	*
Most common diagnosis	7% referred to exclude a DVT	N/A – all different diagnoses	5% attended respectively with abdominal pain and a head injury
	5% referred respectively for superficial venous thrombosis, abdominal pain, pneumonia and chest pain (to exclude acute coronary syndrome)		
			4% attended with chest pain
			3% attended with a fracture

Note: *Data unavailable.

## Discussion

### Main findings

Only 25% of patients attended their GP prior to attending the ED, despite 86% feeling that it was a good practice to make an appointment at [13]. The majority of patients who attended the ED did so due to an injury, although ‘abdominal pain’ was the most common specific presentation. Over one third of patients who attended the ED were admitted (*n* = 80). A statistically significant higher proportion (49%) of patients that had been referred to the ED by their GP were admitted vs. 33% who self-presented. In addition 32% of those who were referred by their GP received ED specific treatment.

## Findings compared with previous studies

Only one quarter of the patients who attended the ED had been seen by a GP prior to their attendance to the ED, less than the 57% detailed in one study [7]. However, it is unclear if some patients had attempted to arrange an appointment prior to attending the ED and had been unsuccessful, although the latest GP patient survey practice report would suggest that the majority of patients did not find this to be a problem normally and daily emergency appointments were offered and not always fully utilised [13]. Some patients may however be still unaware that they can ring up for emergency appointments or have previously been unable to get one so have not tried again. Therefore it does not remain entirely clear as to why patients did not contact their GP and more research is required into patient’s health beliefs relating to emergency care.

The most common reason for attendance to the ED found was injury, although ‘abdominal pain’ was the most common specific presentation; this correlates with findings in similar studies were trauma and accidents contributed 35.5% of attendances and 61% respectively [18,19]. We found that 36% of patients were admitted to hospital on attending the ED, higher than the 26% seen within the UK between 2012 and 2013 [20]. Of those seen by a GP prior to attendance, 49% were admitted and an additional 32% received ED only treatment. This suggests that GPs can accurately triage the majority of patients into those who are acutely unwell and those who require further investigation or specialist review, although there will be a percentage who GPs may feel they have to exclude a worrying diagnosis even if they believe it is unlikely (for example rashes in acutely unwell children referred to exclude meningitis).

It is interesting that 13 patients who had been seen by a GP about the same presenting complaint still decided to attend the ED about the same problem, two on the same day and one three weeks later, with the remainder between this time (including three one day before). Whilst GPs educate patients on red flag symptoms and often state to attend the ED if any of these symptoms were to occur, it is not always clear why patients feel ED is the next stage even if there is not a dramatic change in their symptoms. 48% of the patients in our study were seen out of hours. It is not clear whether this was because of the urgency of the presentation or because of patient convenience and this could be investigated further in the future.

## Strengths and limitations of the study

This study provides an insight into the medical reasons why patients attended the ED from one general practice surgery over the course of a year where high ED attendances have received a lot of media attention. It also provides information regarding their outcome i.e. whether they are discharged or admitted. It demonstrates that a higher percentage of patients who have seen their GP prior to admission are admitted to hospital.

One of the study`s limitations was that the data did not include the patients that saw a GP and as a result did not attend the ED. Therefore, we cannot comment on whether seeing a GP first may significantly reduce ED attendance. Studies seem to suggest that patient concern and anxiety is a considerable influencing factor and that perhaps attending the ED alleviates this worry more than a GP [21]. Some patients also believe that attending the ED department is more appropriate or ‘better’ than the GP or that the GP would send them to ED anyway or just do not want to wait [18,21]. Others report not wanting ‘to bother their GP’ and not knowing if their GP was available [18,19]. One study showed that 20% of patients would have changed their decision about attending the ED if they had known more about other services available [19].

A key limitation is that this study is only within one general practice which limits its generalisability. The study could be expanded to include a larger population. It would be useful to establish whether the opening hours of general practice influence the overall ED attendance rates; patients may know, for example, that the practice is shut on a Wednesday afternoon and therefore choose to attend the ED and future research could attempt to establish if there are correlations between practice closures and ED attendances. Also, this was a retrospective study and relied on correct Read codes being inputted into SystmOne following the receipt of a discharge summary from the hospital. Whilst all emergency admission and attendance codes were searched for and checked, a prospective study would ensure no cases were missed.

Patient beliefs and previous experience may influence their behaviours. Further research is required to explore the relationship between health beliefs and past experiences of healthcare services and choice of providers. However, there is some evidence that ‘inappropriate ED attenders’ tend to be patients who utilise primary care more [8].

## Conclusion

Only 25% patients had attended their GP prior to attendance at the ED, despite emergency appointments being offered Monday to Friday. A higher proportion of those admitted had been seen by their GP and referred in to the emergency department, suggesting the GPs often recognise when admission is required. It remains unclear why the majority of patients did not contact their GP prior to attending the ED, despite daily emergency appointments being offered, and research into patients’ health beliefs are required, alongside evaluation of their previous experience related to ED attendances. More prospective work is also required to establish the associations between visiting a GP and the prevention of ED attendance to determine if access to general practice is the solution for rising ED admissions.

## Disclosure statement

No potential conflict of interest was reported by the authors.
